# *Bdh2* Deficiency Promotes Endoderm-Biased Early Differentiation of Mouse Embryonic Stem Cells

**DOI:** 10.3389/fcell.2021.655145

**Published:** 2021-04-08

**Authors:** Yuting Fu, Fangyuan Liu, Shuo Cao, Jia Zhang, Huizhi Wang, Baojiang Wu, Yongli Song, Shuguang Duo, Xihe Li, Siqin Bao

**Affiliations:** ^1^State Key Laboratory of Reproductive Regulation and Breeding of Grassland Livestock, Inner Mongolia University, Hohhot, China; ^2^Institute of Animal Genetic Research of Mongolia Plateau, College of Life Sciences, Inner Mongolia University, Hohhot, China; ^3^Institute of Zoology, Chinese Academy of Sciences, Beijing, China; ^4^Inner Mongolia Saikexing Institute of Breeding and Reproductive Biotechnology in Domestic Animal, Hohhot, China

**Keywords:** BDH2, embryonic stem cells, CRISPR/Cas9, endoderm differentiation, DNA methylation

## Abstract

3-hydroxybutyrate dehydrogenase-2 (*Bdh2*), a short-chain dehydrogenase, catalyzes a rate-limiting step in the biogenesis of the mammalian siderophore, playing a key role in iron homeostasis, energy metabolism and apoptosis. However, the function of *Bdh2* in embryonic stem cells (ESCs) remains unknown. To gain insights into the role of *Bdh2* on pluripotency and cell fate decisions of mouse ESCs, we generated *Bdh2* homozygous knockout lines for both mouse advanced embryonic stem cell (ASC) and ESC using CRISPR/Cas9 genome editing technology. *Bdh2* deficiency in both ASCs and ESCs had no effect on expression of core pluripotent transcription factors and alkaline phosphatase activity, suggesting dispensability of *Bdh2* for self-renewal and pluripotency of ESCs. Interestingly, cells with *Bdh2* deficiency exhibited potency of endoderm differentiation *in vitro*; with upregulated endoderm associated genes revealed by RNA-seq and RT-qPCR. We further demonstrate that *Bdh2* loss inhibited expression of multiple methyltransferases (DNMTs) at both RNA and protein level, suggesting that *Bdh2* may be essentially required to maintain DNA methylation in ASCs and ESCs. Overall, this study provides valuable data and resources for understanding how *Bdh2* regulate earliest cell fate decision and DNA methylation in ASCs/ESCs.

## Introduction

Mouse embryonic stem cells (ESCs), derived from the inner cell mass (ICM) of blastocysts, can be maintained by self-renewal or differentiate into the three germ layers in embryonic tissues (Evans and Kaufman, 1981). Inhibitors of Mek1/2 (PD0325901, PD) and Gsk3β (CHIR99021, CH), enhanced the derivation of ESCs and promoted ground-state pluripotency (Ying et al., 2008), which was defined as “naïve pluripotency” when LIF was added (2i/L-ESCs) (Nichols and Smith, 2009). The balance of ESCs self-renewal and differentiation is tightly regulated and accurately orchestrated through the modulation of chromatin structures associated with transcriptional regulation (Young, 2011; Tee and Reinberg, 2014; Novo et al., 2016). Three germ layers can be acquired *in vitro* by inducing spontaneous and irreversible differentiation through aggregation of stem cells known as embryoid bodies (Posfai et al., 2014; Chen et al., 2016). However, it remains elusive how ESCs exit pluripotency and undergo lineage differentiation.

Guo et al. (2006) identified a novel cytosolic 3-hydroxybutyrate dehydrogenase-2 (*Bdh2*) as a short-chain dehydrogenase/reductase family member, originally named as *DHRS6*. It has been reported *Bdh2* catalyzes a rate-limiting step in the biogenesis of the mammalian siderophore 2, 5-dihydroxybenzoic acid (2, 5-DHBA), which identified as an iron-binding molecule in mammalian cells (Devireddy et al., 2010). In cultured mammalian cells, as well as in developing zebrafish embryos, depletion of the mammalian siderophore by inhibiting expression of *Bdh2* results in abnormal accumulation of intracellular iron and heme deficiency (Devireddy et al., 2010; Davuluri et al., 2016). *Bdh2* null mice developed microcytic anemia and tissue iron overload, especially in the spleen, and exogenous supplementation with 2,5-DHBA alleviates splenic iron overload in *Bdh2* null mice (Liu et al., 2014a). In addition, *Bdh2* can also play a physiological role in ketone body metabolism, tricarboxylic acid cycle and iron-limiting innate immunity (Guo et al., 2006; Zughaier et al., 2014a, b). Furthermore, Yang et al. (2013) reported that *Bdh2* acts as an anti-apoptotic factor, through a caspase-3 independent pathway. As a result, *Bdh2* may be an independent poor prognosis marker of acute myeloid leukemia by affecting apoptosis (Yang et al., 2013). Overall, *Bdh2* may be a multifunctional gene in mammalian cells. However, the function of *Bdh2* in mouse ESCs and the potential mechanisms involved have not been reported.

We recently showed that the advanced embryonic stem cells (ASCs) represent an intermediate state between naïve (ESCs) and primed pluripotency such as epiblast stem cells (EpiSCs) (Bao et al., 2018). We observed a differential level of *Bdh2* expression between ASCs and ESCs. In order to investigate the precise role of *Bdh2* in the differentiation of ESCs, bi-allelic *Bdh2*-knockout ASCs and ESCs were generated by CRISPR/Cas9. Our results reveal that *Bdh2* plays distinct roles in the regulation of primitive endoderm differentiation *in vitro*, despite the pluripotency is maintained in *Bdh2*-knockout ASCs/ESCs. *Bdh2*-knockout ASCs/ESCs display unique molecular features, which implies the function of *Bdh2* in the maintenance of self-renewal, pluripotency and differentiation.

## Materials and Methods

### Cell Culture

Advanced embryonic stem cells and *Bdh2*-knockout ASCs were cultured in ABCL culture medium comprised Activin A (20 ng/mL, R & D systems), BMP4 (50 ng/mL, R & D systems), CHIR99021 (3 μM, Miltenyi Biotec) and leukemia inhibitory factor (1000 U ml^–1^, Millipore) added into basic N2B27 medium including 50% Neurobasal (Gibco), 50% DMEM/F12 (Gibco), 2 mM GlutaMax (Gibco), 1 × non-essential amino acids (NEAA, Gibco), 1 × Penicillin/Streptomycin (Gibco), 0.1 mM β-mercaptoethanol (Gibco), 0.005% (25 mg) BSA (Gibco) supplemented with 0.5 × N2 (Gibco) and 0.5 × B27 (Gibco). ESCs and *Bdh2*-knockout ESCs were cultured in 2i/L medium consisting of basic N2B27 medium supplemented with PD0325901 (1 μM, Miltenyi Biotec), CHIR99021 (3 μM, Miltenyi Biotec) and leukemia inhibitory factor (1000 U mL^–1^, Millipore). Green fluorescence indicated that GFP expression of the reporter was under the control of *Oct4* promoter and distal enhancer. The colonies could stably passage by Accutase (Life technology) regularly at every 2–3 days. All using plates were coated by fibronectin (1 mg/mL in PBS, Millipore) at least 0.5 h before use.

### *Bdh2*-Knockout ASCs/ESCs Production by CRISPR/Cas9 System

The guide RNAs (gRNAs) were designed using CRISPOR^1^ online tool and were synthesized (Sangon Biotech). The sgRNA was cloned PX459 plasmid that was linearized with *Bbs*I restriction enzyme (Thermo Fisher Scientific). Then, plasmids containing sgRNA were transfected into ASCs and ESCs with lipofectamine 2000 (Invitrogen). Transfected cells were treated with 0.6 μg/μL of puromycin at 48 h post-transfection. Then, the selected cells were subjected to fibronectin-coated 96-well plate to obtain a single cell colony. Approximately after 6 days of colony formation, each single colony was picked and expanded. To confirm the specificity of targeting, genomic DNA was extracted from individual clones by TIANamp Genomic DNA Kit (TIANGEN Biotech) and was sequenced.

### AP Staining

Before staining, cells were placed in 4-well plates and washed with 1 × PBS, then fixed in 4% paraformaldehyde at room temperature for 30 min and washed with 1 × PBS again followed by adding AP staining solution. AP staining solution was prepared as following: gently mixed 50 μL sodium nitrite solution with 50 μL FRV-alkaline solution and placed the mixture at 37°C for 3 min, next added 2.25 mL H_2_O and 50 μL naphthol-As-BI alkaline solution into the mixture, finally mixed gently and incubated staining solution with fixed cells in the dark for overnight.

### Karyotype

The tested cells were incubated with 0.2 μg/mL colchicine in culture medium for 2 h and dissociated by using Accutase, then centrifuged at 1500 r/min for 5 min to collect the tested cells. The cells were gently resuspended in 8 ml 0.075 mol/L KCL (Sigma) and incubated in 37°C water bath kettle for 40 min for hypotonic treatment. Stationary liquid (methanol: glacial acetic acid = 3: 1) of 1 mL was subsequently added to the resuspended cells and mixed gently followed by centrifuging at 1000 r/min for 10 min. After discarding supernatant, the cells were mixed gently in 8 mL stationary liquid and incubated in 37°C water bath kettle for 30 min for cell fixation, which repeated twice. Then resuspended the cells with 0.5 mL stationary liquid and dripped the resuspended cells on ice cold glass slides, followed by drying the glass slides for 1 h in 70°C drying oven. The glass slides were stained in Giemsa (Sigma) for 10 min and washed by distilled water, then air-dried following analyzed by LUCIA Cytogenetics.

### RT-qPCR

Total RNA was extracted by Rneasy Mini Kit (Qiagen) and cDNA was isolated by GoScript Reverse Transcription System (Promega). RT-qPCR reactions were set up using the SYBR FAST Universal qPCR kit (KAPA). Relative expression values were normalized to Gapdh expression and data was performed using Ct computing method 2^–ΔΔCt^. Each experiment was performed in technical triplicate. A list of primers used in Supplementary Table 1. Significance between different groups was determined using *T*-test, ^∗^*P* < 0.05, ^∗∗^*P* < 0.01, ^∗∗∗^*P* < 0.001. The sequences of primers used are listed in Supplementary Table 3.

### Western Blot

Cells were plated onto 6-well plates at 3 × 10^5^ per well and incubated. Subconfluent cells (about 6 × 10^6^) were collected with Accutase, washed three times with cold PBS, and lysed in buffer that contained 20 mM Tris (pH 8.0), 137 mM NaCl, 100 g/L glycerol, 50 g/L Triton X-100, and 4 g/L EDTA; 1 μL PMSF (0.1 M) and 10 μL phosphatase inhibitor (10 g/L) were added per 1 mL lysis buffer immediately before use. The cell lysates were put on ice for 15 min and centrifuged at 12,000 rpm at 4°C for 10 min, and the supernatant was transferred to new tubes. The concentrations of the lysates were measured by Coomassie Plus (Bradford) Assay (Thermo Scientific). Equal amounts (40 μg) of protein were electrophoresed on 12% (w/v) sodium dodecylsulfate polyacrylamide gels. Proteins were transferred to Hybond-polyvinylidene difluoride membranes (Amersham) and incubated with the primary antibodies overnight at 4°C and peroxidase-conjugated secondary antibodies at room temperature for 1 h. Enhanced chemiluminescence (ECL) (Amersham) was used to detect the signals.

### Immunofluorescence

Cells for immunofluorescence assays were washed with PBS and fixed in 4% paraformaldehyde for 30 min at room temperature, and following permeabilized with 0.1% Triton X-100 (Sigma) and 1% BSA in PBS for 30 min. Then incubated cells with primary antibody at 4°C overnight. The cells were subsequently washed three times in 1% BSA, 0.1% Triton X-100 in PBS for 5 min per wash, and incubated with secondary antibody for 1 h at room temperature in the dark, then washed once for 5 min in 1% BSA, 0.1% Triton X-100 in PBS and twice for 5 min in PBS. The cells were then mounted in Vectashield with DAPI (Vector Laboratories) The samples were observed by laser microscope (Nikon, Tokyo, Japan). The anti-bodies used are listed in Supplementary Table 5.

### Cell Cycle Analysis

Cells were dissociated into single cells using Accutase and collected using centrifugation. After being incubated with Hochest33342 for 1 hr at 37°C in the dark, the cell suspension was filtered using a cell strainer (FACSAria II, BD Biosciences) to remove large clumps of cells. The cells were then performed flow cytometry (FACSAria II, BD Company). Data analysis was performed using FlowJo software.

### Generation of Chimera

To generate chimeric embryo, donor cells (12∼15 cells/blastocyst) were microinjected into the ICR mice blastocoel cavity using a piezo-assisted micromanipulator attached to an inverted microscope and recover the injected blastocysts in KSOM medium (Millipore), then transplant the chimeric blastocysts into the uterus of pseudopregnant ICR female mice at 2.5 dpc (days post coitus). The chimeric embryos were collected at E13.5.

### Transcriptome Analysis

Total RNA of ASCs and *Bdh2*-knockout ASCs was isolated by Rneasy Mini Kit (Qiagen). cDNA was synthesized from purified RNA templates. The cDNA libraries were sequenced using Illumina HiSeq10×. The sequencing-obtained raw image data were transformed into sequence data, called raw data or raw reads, which were deposited in the GEO^2^ database under the GEO submission number of GSE158256. Raw data was firstly processed to remove reads with more than 20% low-quality bases and to remove adaptors. Then the clean data were mapped to the reference genome (GRCm38/mm10) with Hisat2(v2.0.5) with default settings. FeatureCounts (v1.5.0-p3) was used for reads counting, and then FPKM (Fragments Per Kilobase of transcript sequence per Millions) of each gene was calculated to estimate gene expression levels. DEGs in different samples were determined using the DEseq2 R package (v1.16.1) with fold change ≥ 1 and adj *p*-value ≤ 0.05. Heatmaps of selected genes were plotted by using the pheatmap R package (v1.0.12). GO and KEGG enrichment analysis was implemented by the clusterProfiler R package. Gene Set Variation Analysis was performed by using the GSVA R package (v1.32.0) and limma package (v3.40.6) with fold change ≥ 0.6 and adj *p*-value ≤ 0.05. GSEA analysis was performed with the GSEA software (v4.0.3) developed by the Broad Institute.

### *In vitro* Differentiation of ASCs/ESCs

Cells on fibronectin-coated plate were incubated with Accutase for 3 min at 37°C till the colonies were completely disaggregated. The colonies were resuspended in EB medium: DMEM/F12 + 20% Knockout serum replacement, KSR (Gibco) + 1% GlutaMax (Gibco) + 1% NEAA (Gibco) + 1% β-mercaptoethanol (Gibco). The colonies were cultured in suspension for up to 7 days at a concentration of 1 × 10^5^ cells/mL.

For primitive endoderm (PrE) differentiation, ASCs and ESCs were differentiated to naïve PrE over the course of 4–8 days by plating 3.7–5.3 × 10^4^ cells cm^–2^ onto gelatinized plates in ESC medium for 24 h. And then cells were grown in RPMI 1640 plus Glutamax (Gibco, 61870036) supplemented with 1 μM Retinoic Acid (Sigma), 3 μM CHIR99021, 20 ng/mL Activin A and 10 ng/mL leukemia inhibitory factor. The medium was changed daily. ESCs differentiated to PrE cells within 4–8 days depending on the cell line and conditions.

### Statistical Analysis

Descriptive statistics were generated for all quantitative data, expressed as mean ± SD. The mean ± SD values were calculated from three samples per group and three technical replicas for per samples. Differences in means between control and *Bdh2*-knockout groups were determined by Student’s *T*-test. All statistical analysis were performed using GraphPad Prism, v.5.0 (GraphPad Software, CA, United States).

### Ethics Statement

All animal experiments were performed in accordance with the National Research Council Guide for the Care and Use of Laboratory Animals and were approved by the Institutional Animal Care and Use Committee at Inner Mongolia University, China.

## Results

### *Bdh2* Is Distinctly Expressed in Different State Mouse Embryotic Stem Cells

Given that *Bdh2* is known to regulate iron homeostasis, energy metabolism and apoptosis (Yang et al., 2013; Liang et al., 2019; Liu et al., 2014b, 2020), we hypothesized that *Bdh2* may play a role in the regulation of self-renewal, pluripotency or differentiation in ESCs. To investigate these questions, we first analyzed its expression across different state mouse ESC lines in previously published RNAseq datasets of ESC, EpiSC and ASC (Bao et al., 2018). ASCs represents an intermediate state between naïve ESC and primed pluripotency such as EpiSC, which is highly potent and has higher levels of DNA methylation (Bao et al., 2018; Wu et al., 2020). We observed *Bdh2* was expressed at lower levels in ground/naïve state ESCs; in contrast, higher level expression was seen in advanced ESCs with the highest levels in ASCs (Supplementary Figure 1A). To confirm this finding, both RT-qPCR and Western-blot analysis were performed and showed that *Bdh2* expression was significantly higher in ASCs compared with ESCs (Supplementary Figures 1B,C).

### Generation of Bi-allelic *Bdh2*-Knockout Mouse Advanced Pluripotent Embryonic Stem Cells (ASCs) and Embryonic Stem Cells (ESCs)

**FIGURE 1:**
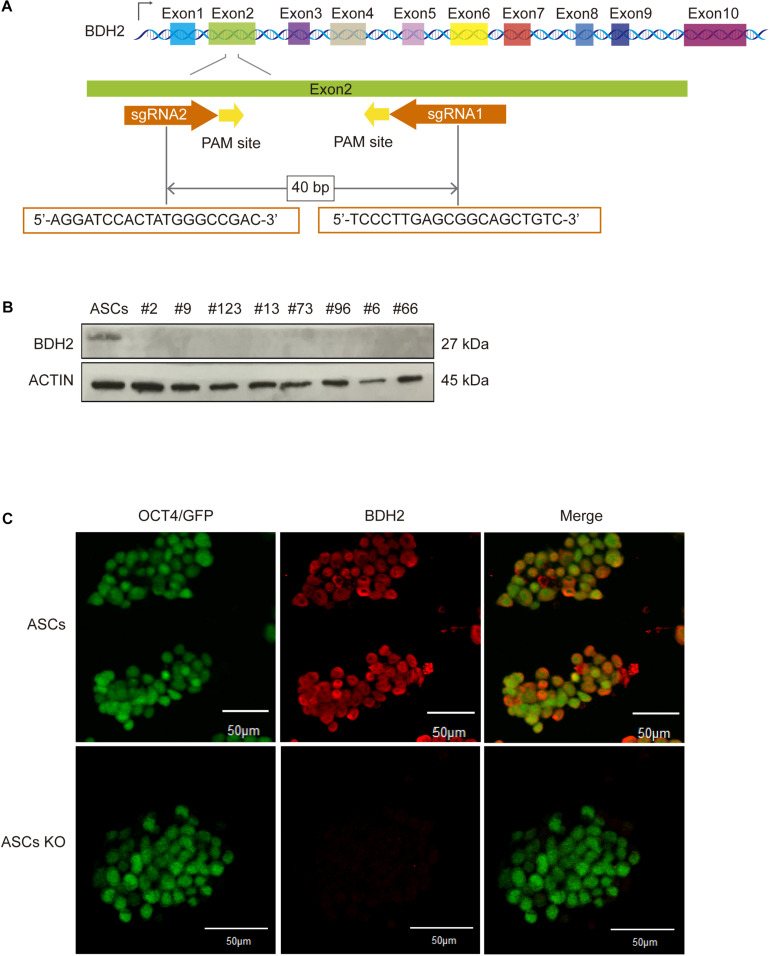
Generation and identification of bi-allelic *Bdh2*-knockout mouse advanced pluripotent stem cells (ASCs). **(A)** Schematic of the exon of *Bdh2* gene and location of gRNAs. **(B)** Western blotting was used to detect the expression of BDH2 in the above eight knockout cells. **(C)** Immunostaining for BDH2 in ASCs and bi-allelic 56-nt clone. nt, nucleotide; PCR, polymerase chain reaction. Scale bars, 50 μm.

CRISPR/Cas9 system was applied to target the CDS1 of the *Bdh2* gene using two sgRNAs near the ATG codon (Figure 1A). The sequence of two sgRNAs was listed in Table S1. A total of 98 ASCs and 101 ESCs single colonies were picked for characterization of editing events. PCR was performed, and electrophoresis and Sanger sequencing of PCR products revealed eight ASCs and two ESCs that carried nucleotide deletions. The sequence of primers used in the PCR was listed in Supplementary Table 2. Out of the eight ASC lines, seven were *Bdh2*^–/–^ and one was *Bdh2*^+/–^; all the two ESC lines were *Bdh2*^–/–^ (Supplementary Figures 1D–F). One of the ASCs possessed a bi-allelic 56-nucleotide (nt) deletion, as well as one of the ESCs possessed a bi-allelic 46-nt deletion (Supplementary Figures 1G,H). The establishment rate of *Bdh2*-knockout ASCs and ESCs were 8.2 and 2%, respectively (Supplementary Table 3).

The deletions generated in the ASC and ESC lines are frameshift mutations which result in a premature stop codon. Several studies indicate that mRNAs with premature stop codon are eliminated in the cells by nonsense-mediated mRNA decay pathway (Danckwardt et al., 2002; Frischmeyer et al., 2002). To verify whether *Bdh2* was not expressed in these edited ASC and ESC lines, RT-qPCR, western blot and Immunostaining were employed. The results showed that *Bdh2* mRNAs were knocked down to 10–40% in the eight *Bdh2*-knockout ASCs (Supplementary Figure 1I). Importantly, BDH2 protein was barely detectable in all the *Bdh2*- knockout ASCs (Figures 1B,C). Similarly, *Bdh2* mRNAs were also knocked down to 30-50% in two *Bdh2*- knockout ESCs (Supplementary Figure 1J), and the protein levels of BDH2 were undetectable in the two *Bdh2*-knockout ESCs (Supplementary Figures 1K,L).

Taken together, multiple *Bdh2*-knockout ASC and ESC lines were generated; ASCs with the bi-allelic 56 nt-deletion and ESCs with bi-allelic 46 nt-deletion were selected for further studies, in which the *Bdh2* protein translation was successfully abolished.

### *Bdh2* Deficient ASCs and ESCs Display Distinct Cellular Properties in Proliferation and Differentiation

**FIGURE 2:**
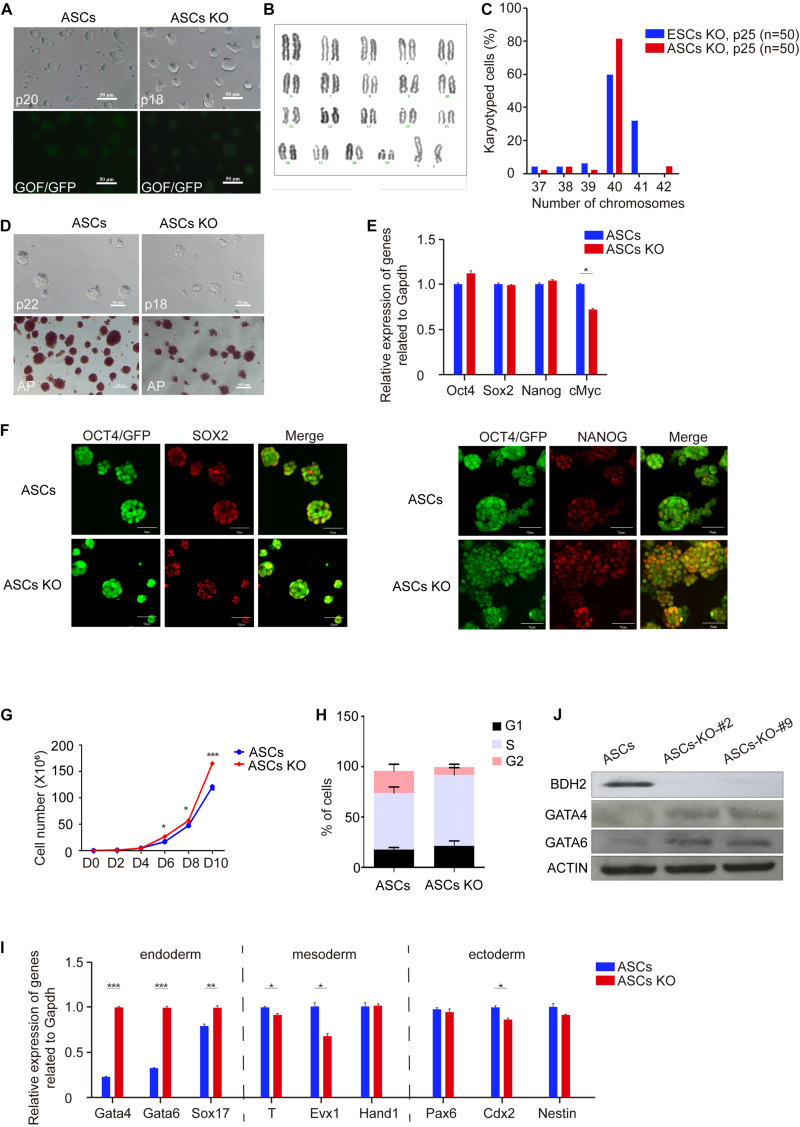
Characteristics of *Bdh2*-knockout ASCs. **(A)** Morphology of *Bdh2*-knockout ASCs. Here, we use ASCs with GOF/GFP reporter. Scale bars, 50 μm. **(B)** Karyotyping of *Bdh2*-knockout ASCs (P25). **(C)** Distribution of chromosome number in *Bdh2*-knockout ASCs (P25) and *Bdh2*-knockout ESCs (P25). **(D)** Alkaline phosphatase (AP) staining on *Bdh2*-knockout ASCs and ASCs. Scale bars, 50 μm. **(E)** Real-time PCR analysis of pluripotency-associated genes expression in the *Bdh2*-knockout ASCs, ASCs were used as control. Data were obtained in triplicate and presented as mean ± SD. *p*-values were calculated by two-way ANOVA, *p < 0.05, **p < 0.001, ***p < 0.0001. **(F)** Immunostaining of SOX2 and NANOG in *Bdh2*-knockout ASCs and ASCs. Scale bars, 50 μm. **(G)** Cell proliferation curves in *Bdh2*-knockout ASCs and ASCs. Data were obtained in triplicate and presented as mean ± SD. *p*-values were calculated by two-way ANOVA, *p < 0.05, **p < 0.001, ***p < 0.0001. **(H)** Cell cycle analysis of *Bdh2*-knockout ASCs and ASCs. The percentage of cells in G1, S, and G2 phase were determined by flow cytometry. Values are means of three independent experiments. Error bars indicate mean ± SD. **(I)** Real-time PCR analysis of endoderm, mesoderm, and ectoderm-associated genes expression in the *Bdh2*-knockout ASCs, ASCs were used as control. Data were obtained in triplicate and presented as mean ± SD. *p*-values were calculated by two-way ANOVA, *p < 0.05, **p < 0.001, ***p < 0.0001. **(J)** Western blotting was used to detect the expression of BDH2, GATA4, and GATA6 in *Bdh2*-knockout ASCs and ASCs.

*Bdh2*-knockout ASCs and ESCs exhibited normal morphology similar to wide type (WT) ASCs and ESCs (Figures 2A–C and Supplementary Figures 2A,B). Both WT and *Bdh2*-knockout ASCs/ESCs were alkaline phosphatase (AP)-positive colonies, indicating that deletion of *Bdh2* had no effect on self-renewal potential of the ESCs (Figure 2D and Supplementary Figure 2C). To understand the characteristics of *Bdh2*-knockout ASCs/ESCs, we next determined the expression levels of several pluripotency markers. Both the mRNA and protein of these genes, OCT4, SOX2, and NANOG, were expressed at similar levels in WT and *Bdh2*-knockout ASCs/ESCs (Figures 2E,F and Supplementary Figures 2D,E). Interestingly, *Bdh2*-knockout ASCs propagated more quickly than ASCs, as shown in cell growth curves (Figure 2G). However, *Bdh2* deficiency did not affect the proliferation of ESCs (Supplementary Figure 2F).

To confirm these findings, we performed cell cycle analysis. ESCs have a unique cell cycle structure, including long S phase and remarkably short G1 and G2 phase (White and Dalton, 2005). The percentages of cells in G1, S, and G2 phase were determined by flow cytometry. As expected, *Bdh2* deficiency promoted cell growth of ASCs as judged by an increase in the percentage of *Bdh2*-knockout ASCs in S phase compared to ASCs (Figure 2H). However, *Bdh2*-knockout has little impact on cell cycle of ESCs (Supplementary Figure 2G). Collectively, these findings were consistent with the cell proliferation results (Figure 2G and Supplementary Figure 2F).

To address whether *Bdh2* deficiency modulates the expression of gene markers for three germ layers, i.e., endoderm, mesoderm and ectoderm, RT-qPCR was performed. Here, the RT-qPCR analysis data showed that the expression of endoderm marker *Gata4*, *Gata6*, and *Sox17* was significantly upregulated in *Bdh2*-knockout ASCs. In contrast, expression of mesoderm genes *Brachyury* (*T*), *Evx1* and trophectoderm gene *Cdx2* was significantly decreased (Figure 2I). Similarly, the expression of *Gata4* and *Gata6* was found to be significantly upregulated in *Bdh2*-knockout ESCs, while *Brachyury* (*T*) and *Cdx2* transcripts significantly was decreased in *Bdh2*-knockout ESCs (Supplementary Figure 2H). These findings were confirmed by Western Blot and Immunoflourance staining against GATA4, GATA6, SOX17 (Figure 2J and Supplementary Figures 2I,J).

Taken together, our results indicate that *Bdh2* deficiency in ASCs and ESCs did not affect the pluripotency and identity of mouse ESCs. Interestingly, *Bdh2* deficiency promoted proliferation of ASCs but not ESCs by affecting the cell cycle phase distribution. More importantly, deletion of *Bdh2* increased the expression of endoderm marker genes while decreasing the expression of some mesoderm and ectoderm genes. These results suggest that *Bdh2* plays distinct roles in regulating stem cell fate.

### RNA-seq Reveals *Bdh2*-Knockout ASCs Display Molecular Features of Endoderm Differentiation and Reduced Metabolic Processes

**FIGURE 3:**
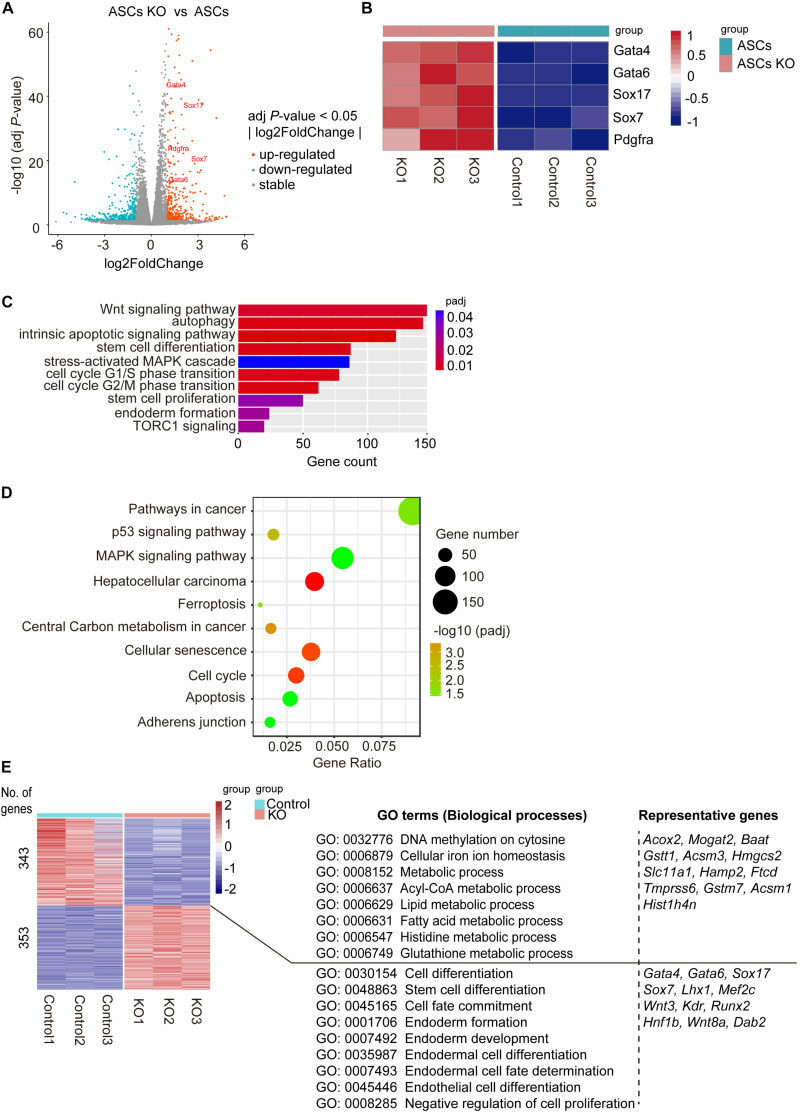
Analysis of molecular features of *Bdh2*-knockout ASCs. **(A)** Volcano plot of differentially expressed genes for *Bdh2*-knockout ASCs versus ASCs. **(B)** Heatmap showing scaled expression of endoderm-associated genes. **(C)** Gene Ontology (GO) analysis of significantly enriched “biological processes” (BP) of the differentially expressed genes (DEGs) for *Bdh2*-knockout ASCs versus ASCs described in **(A)**. **(D)** Kyoto Encyclopedia of Genes and Genomes (KEGG) pathway analysis of DEGs for *Bdh2*-knockout ASCs versus ASCs described in **(A)**. **(E)** Heatmap showing scaled expression values of a total of 696 DEGs [mean log2 (normalized read counts) > 2, log2 (fold change) ≥ 1, adjusted *p* value < 0.05] in *Bdh2*-knockout ASCs versus ASCs. Significantly enriched GO terms and representative genes in each cluster are listed on the right.

To gain further molecular insights into *Bdh2*-knockout ASCs, we determined global gene expression profiles using RNA sequencing (RNA-Seq). To examine differences between *Bdh2*-knockout ASCs and WT ASCs, we analyzed differentially expressed genes using *DEseq2 R package* (adj *p*-value < 0.05, fold change |log2Ratio| ≥ 0). There were 5673 differentially expressed genes in *Bdh2*-knockout ASCs compared with WT ASCs; 3175 genes were significantly upregulated, including major endoderm lineage markers (*Gata4*, *Gata6*, *Sox17*, *Sox7*, *Pdgfra*), and 2498 genes were significantly downregulated which are mainly related to metabolic processes (Figure 3A). It is interesting to note that the primitive endoderm markers (*Gata4*, *Gata6*, *Sox7*) and endoderm transcription factor genes (*Sox17*) were highly expressed in *Bdh2*-knockout ASCs compared to WT ASCs (Figure 3B), in line with the RT-qPCR and Western Blot results. Together, these data suggest that *Bdh2* may influence the early differentiation of ESCs.

Genes ontology (GO) analysis suggested that GO terms such as “stem cell differentiation,” “endoderm formation,” “stem cell proliferation,” “cell cycle,” “autophagy,” “intrinsic apoptotic signaling pathway” and a number of metabolic process terms in biological process were enriched in the differentially expressed genes between *Bdh2*-knockout ASCs and WT ASCs (Figure 3C and Supplementary Figure 3A). Kyoto Encyclopedia of Genes and Genomes (KEGG) pathway enrichment analysis showed the top 10 enriched pathways (Figure 3D), including “ferroptosis,” “cell cycle,” “apoptosis,” “adherens junction,” “pathways in cancer,” *etc*. When cutoff of adj *p*-value < 0.05, fold change |log2Ratio| ≥ 1 was applied, there were 696 differentially expressed genes. Upregulated genes were mainly enriched in such “GO-biological processes” as “negative regulation of cell proliferation,” “cell fate commitment,” and “endoderm formation,” with representative genes as *Gata4*, *Gata6*, *Sox17*, *Sox7*, *Wnt3*, *Runx2*. The downregulated genes were primarily enriched in DNA methylation (“DNA methylation on cytosine”) and metabolism (“cellular iron ion homeostasis,” “Lipid metabolic process,” “histidine metabolic process,” “glutathione metabolic process,” and “Acyl-CoA metabolic process”), with representative genes as *Acox2*, *Mogat2*, *Gsttl1*, *Ftcd*, *Histlh4n* (Figure 3E and Supplementary Figure 3B).

In line with the GO analysis, GSEA analysis revealed enrichment for cell fate specification and several metabolic processes (Supplementary Figure 3C). It is worth noting that adhesion-related genes, enriched in such GO terms as “adherens junction,” “focal adhesion,” and “gap junction,” were all down-regulated in *Bdh2*-knockout ASCs (Supplementary Figure 3D). Taken together, *Bdh2* may regulate adhesion of ASCs.

The RT-qPCR was performed on key genes and showed high concordance with the RNA-Seq data (Figure 2I and Supplementary Figure 2H). Overall, these results showed that the knockout of *Bdh2* altered the ASCs with particular molecular features. In short, the endoderm lineage markers were significantly upregulated, while the genes associated with various metabolic processes were significantly downregulated in *Bdh2*-knockout ASCs.

### *Bdh2* Deficiency Affects DNA Methylation in ASCs/ESCs

**FIGURE 4:**
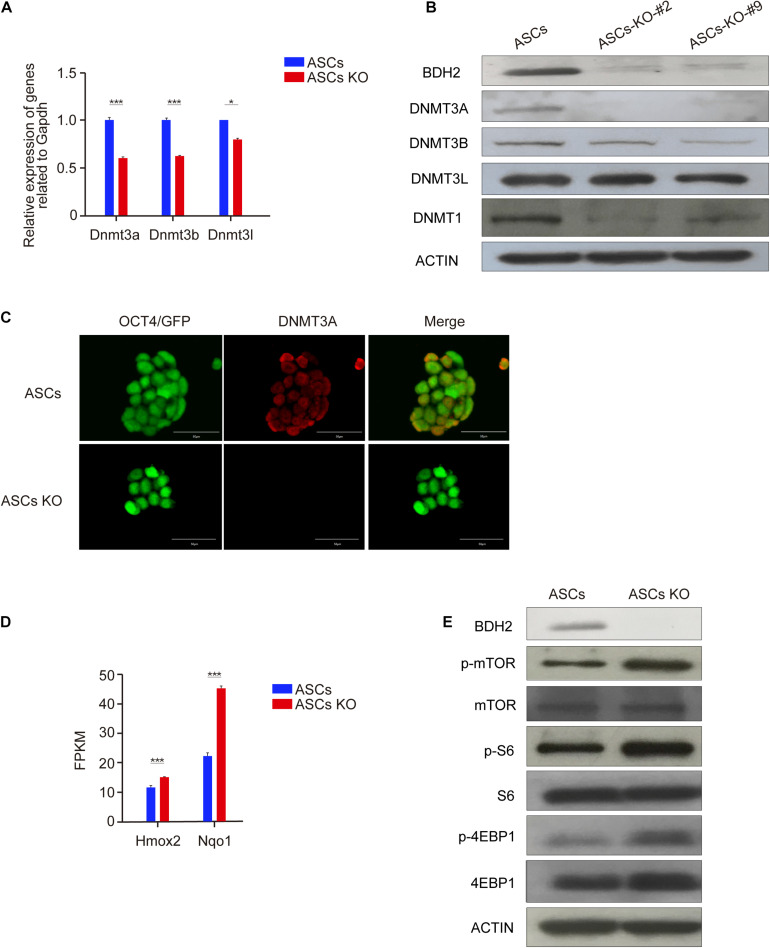
*Bdh2* knockout regulates DNA methylation in ASCs. **(A)** Real-time PCR analysis of DNA methyltransferase genes expression (Dnmt3a, Dnmt3b, and Dnmt3l) in the *Bdh2*-knockout ASCs, ASCs were used as control. Data were obtained in triplicate and presented as mean ± SD. *p*-values were calculated by two-way ANOVA, *p < 0.05, ***p < 0.001. **(B)** Western blotting analysis for BDH2, DNMT3A, DNMT3B, DNMT3L, and DNMT1 in two *Bdh2*-knockout ASCs. **(C)** Immunostaining of DNMT3A in *Bdh2*-knockout ASCs and ASCs. Scale bars, 50 μm. **(D)** Expression levels of oxidative stress-related key genes were tested by RNA-Seq analysis. Data were obtained in triplicate and presented as mean ± SD. *p*-values were calculated by two-way ANOVA, *p < 0.05, ***p < 0.001. **(E)** The effect of *Bdh2* knockout on mTOR signaling pathway in ASCs was detected by Western blotting.

It was previously reported that ASCs have higher DNA methylation levels compared with ESCs (Bao et al., 2018; Wu et al., 2020). To investigate if loss of *Bdh2* altered the DNA methylation levels, we examined the mRNA and protein levels of methyltransferases in *Bdh2*-knockout ASCs/ESCs and WT ASCs/ESCs. RT-qPCR analysis demonstrated that *Dnmt3a*, *Dnmt3b*, and *Dnmt3l* were significantly decreased in *Bdh2*-knockout ASCs/ESCs compared to WT ASCs/ESCs (Figure 4A and Supplementary Figure 4A). Furthermore, the protein expression of DNMT3A and DNMT3B by Western Blot were undetectable or much lower in the *Bdh2* knockout lines, consistent with the results of the transcript levels (Figure 4B and Supplementary Figure 4B), however DNMT3L protein levels were comparable. In line with DNMT3A and DNMT3B, the expression of DNA methyltransferases, DNMT1, was also significantly markedly decreased in *Bdh2*-knockout ASCs/ESCs (Figure 4B and Supplementary Figure 4B). Immunofluorescence staining of DNMT3A also confirmed its downregulation in *Bdh2*-knockout ASCs/ESCs as well (Figure 4C and Supplementary Figure 4C).

Zhang et al. (2017) previously found that DNA demethylation was mediated by activation of mTORC1 signaling via inhibiting DNMT1 and DNMT3A expression. Interestingly, mTORC1 signaling can be activated by oxidative stress (Liu et al., 2014c). Given that that *Bdh2* deficiency was reported to increase oxidative stress (Liu et al., 2014c), we speculated that the decreased expression of DNA methyltransferases was related to increased oxidative stress and mTORC1 signaling activity. In support of this notion, RNA-Seq analysis revealed that oxidative stress-related genes, heme oxygenase 2 (*Hmox2*) and NAD(P)H dehydrogenase quinone 1 (*Nqo1*), were significantly upregulated in *Bdh2*-knockout ASCs (Figure 4D). As expected, Western Blot analysis showed that mTORC1 signaling was increased in *Bdh2*-knockout ASCs compared with WT ASCs, in particular the phosphorylation of mTOR protein and its downstream effector proteins, p-S6 and p-4EBP1 (Figure 4E).

Taken together, these results indicate that loss of BDH2 suppress expression of multiple methyltransferases in ASCs, and increased phosphorylation of mTOR and several of the downstream effectors.

### Differential Potency of *Bdh2*-Knockout ASCs/ESCs

To address if *Bdh2* knockout impacts differentiation of ASCs/ESCs *in vitro*, we assessed embryoid body (EB) formation using WT and *Bdh2*-knockout ASC/ESC lines. Suspension culture allows for the formation of cell clusters during spontaneous differentiation (or embryoid bodies; EBs), and can mimic cellular interactions reminiscent of development processes *in vivo* (Boheler et al., 2002).

**FIGURE 5:**
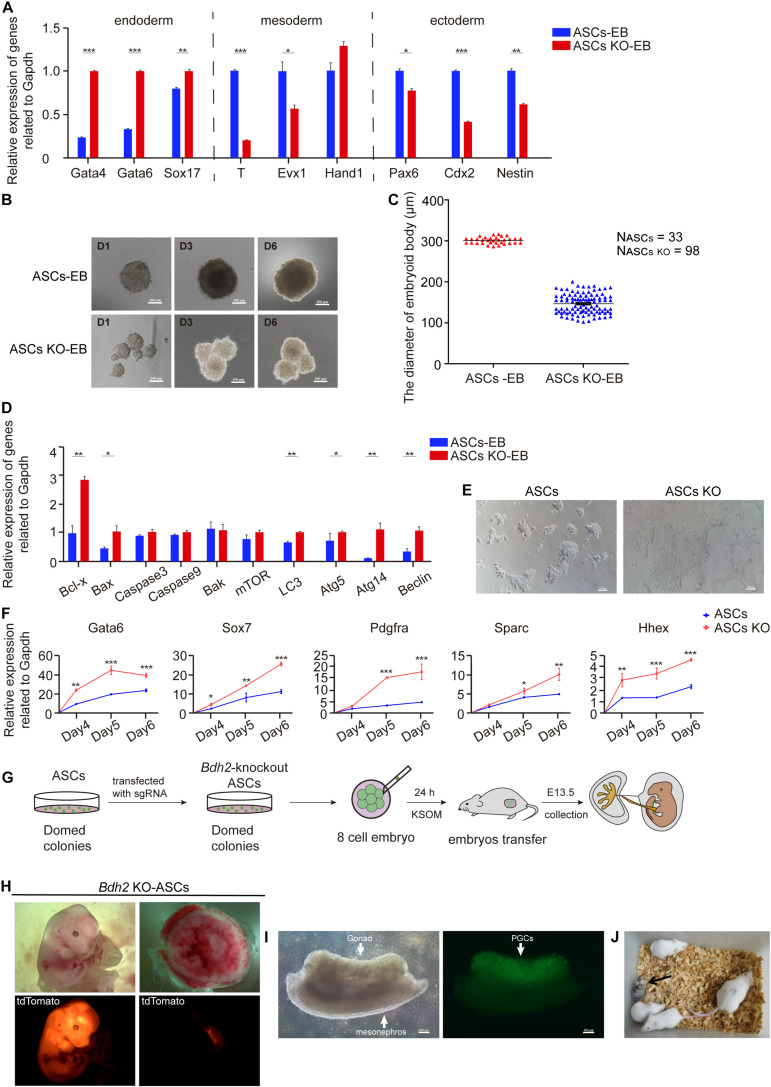
Differentiated and developmental potency of *Bdh2*-knockout ASCs. **(A)** Real-time PCR analysis of endoderm, mesoderm and ectoderm-associated genes expression in the embryoid bodies of *Bdh2*-knockout ASCs, embryoid bodies of ASCs were used as control. Data were obtained in triplicate and presented as mean ± SD. *p*-values were calculated by two-way ANOVA, *p < 0.05, **p < 0.001, ***p < 0.0001. **(B)** Morphology of embryoid bodies induction by *Bdh2*-knockout ASCs and ASCs in Day1, Day3, and Day6. Scale bars, 100 μm. **(C)** Statistics of the number and diameter of embryoid bodies formed by *Bdh2*-knockout ASCs and ASCs, respectively. N, represents the number of EB spheres. **(D)** Real-time PCR analysis of apoptotic and autophagy-associated genes expression in the embryoid bodies of *Bdh2*-knockout ASCs, embryoid bodies of ASCs were used as control. Data were obtained in triplicate and presented as mean ± SD. *p*-values were calculated by two-way ANOVA, *p < 0.05, **p < 0.001, ***p < 0.0001. **(E)** Representative bright-field images from *Bdh2*-knockout ASCs and ASCs differentiated for 5 days toward the primitive endoderm lineage. Scale bars, 50 μm. **(F)** Real-time PCR analysis of key markers after 4/5/6 days of directed differentiation toward primitive endoderm. Data were obtained in triplicate and presented as mean ± SD. *p*-values were calculated by two-way ANOVA, *p < 0.05, **p < 0.001, ***p < 0.0001. **(G)** Schematic illustrating chimera production from *Bdh2*-knockout ASCs. **(H)** Chimeras (E13.5) generated with *Bdh2*-knockout ASCs (multiple cells). ASCs carried tdTomato^+^/GOF^+^ reporter. **(I)** Germline transmission of *Bdh2*-knockout ASCs in E13.5 chimeras. PGCs were shown by GOF/GFP^+^ cells (arrow). Scale bars, 100 μm. **(J)** A chimeric pup generated by injecting 15∼20 *Bdh2*-knockout ASCs in ICR host blastocysts.

RT-qPCR analysis of trilineage markers showed the endoderm markers *Gata4*, *Gata6* and *Sox17* increased in the early stages of *Bdh2*-knockout ASC derived EBs. In contrast the mesodermal markers, *Brachyury* (*T*) and *Evx1* were downregulated in *Bdh2* deficient cells compared with WT cells. Similarly, the ectodermal markers, *Pax6*, *Cdx2*, and *Nestin*, were also downregulated in *Bdh2*-knockout EBs (Figure 5A). We also assessed EBs derived from ESCs, and observed a similar trend (Supplementary Figure 5A). Collectively, our data showed that absence of *Bdh2* in ASCs/ESCs potentiate exit from the pluripotency state and differentiate into derivatives of the three germ layers with bias toward endoderm with compromised formation or the maintenance of mesoderm and ectoderm.

In addition to the gene expression differences, *Bdh2*-knockout EBs presented with a smaller size but a larger number as early as day 2 when compared to the one spherical EBs derived from WT ASCs/ESCs (Figure 5B and Supplementary Figure 5B,C). To further investigate this difference, pendant-drop method was used to form embryoid body. While the WT ASCs/ESCs only formed one EB sphere per drop, there were three times more in the number of EBs derived from *Bdh2*-knockout ASCs (Figure 5C and Supplementary Figure 5D). The diameter of EBs derived from *Bdh2*-knockout ASCs was around 150 μm at day 5, while the diameter of EBs derived from WT ASCs was around 300 μm (Figure 5C). Similarly, the number of EBs derived from *Bdh2*-knockout ESCs was about five times as much as the number of EBs derived from WT ESCs. The diameter of EBs derived from *Bdh2*-knockout ESCs was around 100 μm at day 5, while the diameter of EBs derived from WT ESCs was around 300 μm (Supplementary Figure 5D). More importantly, *Bdh2*-knockout EB spheres seem to be more incompact, which may due to the down-regulation of adhesion-related genes in *Bdh2*-knockout cells (Supplementary Figure 3D).

Interestingly, when *Bdh2*-knockout ASCs/ESCs were differentiated *in vitro*, we observed a striking increase of cell apoptosis at day 3 of differentiation morphologically. We therefore measured the mRNA levels of apoptosis and autophagy-associated genes by RT-qPCR. The results showed that *Bax*, *Bcl-x*, *LC3*, *Atg5*, *Atg14*, and *Beclin* in *Bdh2*-knockout EBs were significantly increased compared to the WT ASCs/ESCs EBs (Figure 5D and Supplementary Figure 5E), and suggest that cells from *Bdh2*-knockout ASCs/ESCs EBs were markedly more sensitive to apoptosis. These results are in agreement with the findings of Yang et al. (2013) that *Bdh2* acts as an anti-apoptotic factor.

### Primitive Endoderm Differential Potency of *Bdh2*-Knockout ASCs/ESCs

We then decided to probe the potential of *Bdh2*-knockout ASCs/ESCs for a different cell fate controlled by inductive cues. Smith demonstrated mESCs do not generate endoderm cells directly, as only late epiblast cells have this capacity. As a result, germ-layer specification can be only attained after transition through different pluripotent states (Smith, 2017). mESCs, however, can directly give rise to primitive endoderm. Thus, we decided to test the capacity of *Bdh2*-knockout ASCs/ESCs for this differentiation by utilizing a protocol based on defined culture conditions (Anderson et al., 2017; Ortmann et al., 2020). The differentiation efficiency was quantified by measuring the expression of PrE markers, *Gata6*, *Sox7*, *Pdgfr*α, *Sparc*, and *Hhex*. Interestingly, *Bdh2*-knockout ASCs/ESCs were more able to produce cells with distinctive morphology expressing *Gata6*, *Sox7*, *Pdgfr*α, *Sparc*, and *Hhex* (Figures 5E,F and Supplementary Figures 5F,G) compared to WT ASCs/ESCs. These analyses confirm that *Bdh2* deficiency influence the capacity of mESCs to directly generate differentiated progenies.

### Developmental Potency of *Bdh2*-Knockout ASCs

To further evaluate the role of *Bdh2* in mESC differentiation, we set to investigate the identity and developmental potential of *Bdh2*-knockout ASCs *in vivo*. *Bdh2*-knockout ASCs were transfected with H2B tdTomato plasmid to perform chimera test by injecting them into 8-cell stage mouse embryos, and the chimeric embryos were transplanted into 2.5 dpc pseudopregnancy female mice (Figure 5G). We analyzed their contribution to chimeric embryos at E13.5 and found that tdTomato-positive *Bdh2*-knockout ASCs contributed robustly to the embryo, and slightly contributed to the yolk sac, as well as placental labyrinth (Figure 5H). Furthermore, *Bdh2*-knockout ASCs were able to contribute to germline lineages (Figure 5I) and full-term chimeras (Figure 5J). These data suggest that the *Bdh2*-knockout ASCs have the pluripotential to contribute to embryo, and generate full-term pups.

Taken together, our data demonstrate that loss of *Bdh2* did not affect the pluripotency of ASCs *in vivo* and *Bdh2* deficient ASCs have the capacity to chimeric mice and contribute to germline lineages. However, analysis of germ layer formation *in vitro* suggests that the *Bdh2* deficiency promoted a biased differentiation toward endoderm.

## Discussion

**FIGURE 6:**
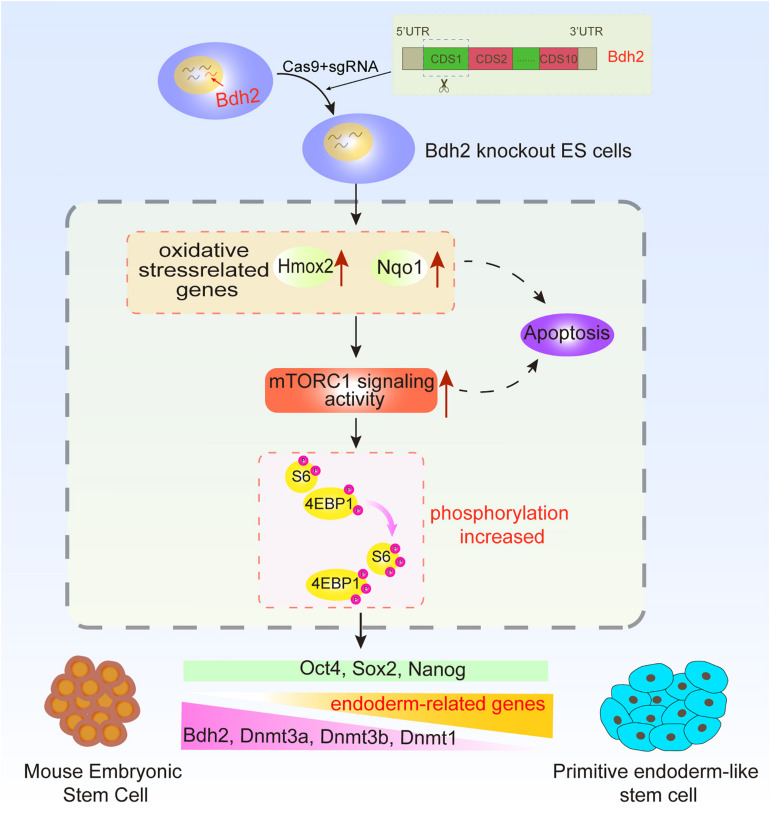
Summary diagram.

In this study, we generated *Bdh2-*knockout ASCs and ESCs using CRISPR/Cas9 system and analyzed the molecular and cellular consequences of *Bdh2* deficiency in ASCs and ESCs. Our results demonstrate *Bdh2*-knockout ASCs/ESCs were capable to maintain pluripotency and contribute to chimeric embryos. Interestingly, *Bdh2* deficiency promoted ASCs/ESCs to a biased differentiation toward primitive endoderm lineage. We demonstrate that loss of *Bdh2* suppressed expression of multiple methyltransferase such as DNMT1, DNMT3A and DNMT3B, together with increased phosphorylation of mTOR and multiple effectors (Figure 6).

In here, we used two types of ESCs with distinct states: one was ASCs generated by our lab (Bao et al., 2018; Wu et al., 2020), which is an advanced state with a unique molecular feature and developmental potential; another was ground state-2i/L-ESCs. In here, we found *Bdh2*-knochout ASCs and ESCs showed typical morphology of WT ASCs/ESCs, and expressed pluripotent genes *Oct4*, *Sox2*, and *Nanog*. These data indicated that *Bdh2* is not essential to maintain pluripotency. Notably, *Bdh2*-knockout ASCs showed increased proliferation with larger fraction of cells in S-phase compared to WT ASCs. We noticed that the percentage of cells in G1 phase appears to be increased in *Bdh2*-knockout ASCs compared to ASCs, as well as with the long S phase (Figure 2H). Soufi and Dalton reviewed that there exists a strong connection between the cell cycle and mechanisms required for executing cell fate decisions, and that G1 phase represents a period of differentiation competency (Soufi and Dalton, 2016). As pluripotent stem cells commit to one of the three embryonic germ layers, their progeny acquire an extended G1 phase, resulting in increased cell division times. The endoderm and mesoderm commitment occur in early G1 phase (Soufi and Dalton, 2016), thus, the increased G1 phase in *Bdh2*-KO cells may explain at least partly, their biased differentiation toward endoderm lineage (Figure 2G). Interestingly, both *Bdh2* deficient ESCs and ASCs showed increased expression of endoderm genes such as *Gata4*, *Gata6*, and *Sox17* in both mRNA and protein level, and decreased expression of several mesoderm and ectoderm related genes such as *Brachyury* (*T*), *Evx1* and *Cdx2*. These data suggest that *Bdh2* is involved in the proper control of germ layer specific transcripts for cell fate decision. Wnt and Nodal signaling play a critical role in endoderm formation (Scheibner et al., 2019), our study show that Wnt signaling was the top pathway enriched in the *Bdh2*-deficient ASCs; it would be interesting to investigate how *Bdh2* regulates Wnt signaling, which in turn controls cell fate decision during germ layer differentiation.

Differential methylation levels are critically associated with establishment and maintenance of the pluripotent state, as well as in the differentiation process in ESCs (Petell et al., 2016; McLaughlin et al., 2019; van Mierlo et al., 2019), methylation regulation has been an area of intense investigation in ESC biology. Our data demonstrate that *Bdh2* deficient ASCs showed markedly reduced expression of multiple methytransferases such as DNMT1, DNMT3A, and DNMT3B suggesting loss of *Bdh2* may cause hypomethylation in ESCs. Recently, Zhao et al. (2018) found that knockdown of *Bdh2* expression caused increased DNA hydroxymethylation and decreased DNA methylation in CD4^+^ T cells, proposing the association between *Bdh2* and DNA demethylation in the pathogenesis of systemic lupus erythematosus, and they revealed that the *Bdh2* knockdown induced DNA hypomethylation was associated with increasing intracellular iron in CD4^+^ T cells. However, from multiple studies, it was suggested that DNA hypomethylation induced by *Bdh2* deficiency can be caused by increased oxidative stress, and consequent activation of mTORC1 signaling, thereby mediating DNA hypomethylation via inhibiting the expression of DNMT1 and DNMT3A (Liu et al., 2014c; Zhang et al., 2017). Our results are consistent with this mechanism; knockout of *Bdh2* induced the expression of oxidative related genes, and the activation of mTORC1 signaling. DNA transmethylases (DNMTs), DNMT3A, DNMT3B, and DNMT1, were also nicely downregulated in *Bdh2*-knochout ASCs/ESCs, suggesting *Bdh2* may be essentially required to control DNA methylation in ASCs/ESCs. However, the molecular mechanisms on how *Bdh2* regulates DNA methylation need to be dissected in further detail.

It is noteworthy that morphological differences between EB spheres derived from *Bdh2*-knockout and WT ASCs/ESCs were remarkable; *Bdh2*-knockout EB spheres seemed to be more incompact compared to the WT EBs. We propose two explanations for this observed difference. Firstly, *Bdh2* plays distinct roles in the regulation of primitive endoderm-like stem cell. It was reported that cellular adhesion of primitive endoderm (PrE) showed difference with epiblast (Stanton et al., 2019). Interestingly, we found downregulation of adhesion-related genes in *Bdh2*-knockout cells (Supplementary Figure 3D), which may explain the formation of smaller EBs in *Bdh2*-knockout ASCs/ESCs. Secondly, *Bdh2*-knockout cells were more apoptotic, at least some of the cells. Previous studies have demonstrated that oxidative stress is responsible for triggering apoptosis and autophagy in tumors (Sato et al., 2004; Kaminskyy et al., 2012; Fernandez-Gil et al., 2019; Zhao et al., 2019), and influences numerous biological processes, including development and differentiation (Meier et al., 2000; Fuchs and Steller, 2011). In our study, a striking increase of cell apoptosis was observed at day 3 of *in vitro* differentiation (Figure 5B and Supplementary Figures 5B,C) with much more obvious apoptosis in *Bdh2*-knockout EBs than in WT EBs, which was in line with the report that *Bdh2* acts as an anti-apoptotic factor (Yang et al., 2013). Indeed, apoptosis and autophagy-associated genes such as *Bcl-x*, *Bax*, *LC3*, *Atg5*, *Atg14*, and *Beclin* were significantly increased in *Bdh2*-knockout EBs compared to the WT ASCs/ESCs EBs. Furthermore, activated mTORC1 signaling in *Bdh2*-knockout cells (Figure 4E) may also be responsible for control of cellular status, as mTOR kinase is a key regulator of homeostasis, ribosome synthesis, various metabolic pathways and autophagy which are known to finely regulates the balance between self-renewal and differentiation in stem cells (Wullschleger et al., 2006; Han et al., 2008; Endo et al., 2009).

Indeed, our data found *Bdh2* deficient ASCs/ESCs could contribute to germ line transmission and full-term pup *in vivo*; the biased endoderm differentiation *in vitro* didn’t affect chimeric development. A possible explanation could be that while *Bdh2*-deficient ASCs/ESCs showed increased endoderm markers, they also express mesoderm and ectoderm genes similar to ASCs/ESCs (Figure 2I and Supplementary Figure 2H).

## Conclusion

We generated *Bdh2* deficient ASC and ESC lines and show that loss of *Bdh2* had no significant impact on the maintenance of self-renewal and the pluripotency of ESCs. Interestingly, our data demonstrate that *Bdh2* deficiency affected the formation of EBs and differentiation (primitive endoderm formation) by regulating the expression of related genes. We further demonstrate that *Bdh2* loss inhibited expression of multiple methyltransferases, suggesting *Bdh2* may be essentially required to maintain DNA methylation which is critically associated with the pluripotent state, as well as in the differentiation process in ESCs. We propose that the bi-allelic *Bdh2*-knockout ASC and ESC lines would be a valuable laboratory resource to study the role of *Bdh2* in the development, as well as a worthwhile tool for earliest cell fate decision and DNA methylation studies.

## Data Availability Statement

The datasets presented in this study can be found in online repositories. The names of the repository/repositories and accession number(s) can be found below: https://www.ncbi.nlm.nih.gov/geo/info/linking.html, GSE158256.

## Ethics Statement

All animal experiments were performed in accordance with the National Research Council Guide for the Care and Use of Laboratory Animals and were approved by the Institutional Animal Care and Use Committee at Inner Mongolia University, China.

## Author Contributions

SB and XL conceived and designed the experiments. YF, SC, JZ, and HW performed the experiments. YF and FL analyzed the data. YF wrote the manuscript. SB, BW, and YS revised the manuscript. All the authors contributed to the article and approved the submitted version.

## Conflict of Interest

The authors declare that the research was conducted in the absence of any commercial or financial relationships that could be construed as a potential conflict of interest.

## Footnotes

1 http://crispor.tefor.net/

2 https://www.ncbi.nlm.nih.gov/geo/info/linking.html

## Supplementary Material

Supplementary Figure 1 Identification of bi-allelic *Bdh2*-knockout mouse embryonic stem cells (ESCs). **(A)** Expression of *Bdh2* in different cell lines by RNA-Seq analysis. Data were obtained in triplicate and presented as mean ± SD. *p*-values were calculated by two-way ANOVA, **p* < 0.05, ***p* < 0.001, ****p* < 0.0001. **(B)** Real-time PCR analysis of *Bdh2* expression in ESCs and ASCs. Data were obtained in triplicate and presented as mean ± SD. *p*-values were calculated by two-way ANOVA, **p* < 0.05, ***p* < 0.001, ****p* < 0.0001. **(C)** Western blotting was used to detect the expression of BDH2 in ESCs and ASCs. **(D)** The target region was sequenced to verify deletion in the desired region (**a**: wild type control; **b**: the sequence of bi-allelic knockout; **c**: the sequence of target region before single cell colony formation that showed overlapped pick in the gRNA site). **(E)** Genomic DNA PCR flanking the gRNAs sites from single cell colonies (1 and 12:DNA ladder; 2: Negative control; 3: Deletion not occurred; 4–9 and 11: Bi-allelic knockout; 10: Deletion occurred in one allele). **(F)** Genomic DNA PCR flanking the gRNAs sites from single cell colonies (1 and 6: DNA ladder; 2: Negative control; 3: Deletion not occurred; 4 and 5: Bi-allelic knockout). **(G)** Alignment between ASCs wild type sequence and bi-allelic knockout sequences that showed 56-nt deleted region. **(H)** Alignment between ESCs wild type sequence and bi-allelic knockout sequences that showed 46-nt deleted region. **(I)** Real-time PCR analysis of *Bdh2* expression in the 7 bi-allelic knockout clones and a one-allelic knockout clone. Data were obtained in triplicate and presented as mean ± SD. *p*-values were calculated by two-way ANOVA, **p* < 0.05, ***p* < 0.001, ****p* < 0.0001. **(J)** Real-time PCR analysis of *Bdh2* expression in the 2 bi-allelic knockout clones. Data were obtained in triplicate and presented as mean ± SD. *p*-values were calculated by two-way ANOVA, **p* < 0.05, ***p* < 0.001, ****p* < 0.0001. **(K)** Western blotting was used to detect the expression of BDH2 in the 2 bi-allelic knockout cells. **(L)** Immunostaining for BDH2 in ESCs and bi-allelic 46-nt clone.

Supplementary Figure 2 Characteristics of *Bdh2*-knockout ESCs. **(A)** Morphology of *Bdh2*-knockout ESCs. Here, we use ESCs with GOF/GFP reporter. Scale bars, 50 μm. **(B)** Karyotyping of *Bdh2*-knockout ESCs (P25). **(C)** Alkaline phosphatase (AP) staining on *Bdh2*-knockout ESCs (P18) and ESCs (P22). Scale bars, 50 μm. **(D)** Real-time PCR analysis of pluripotency-associated genes expression in the *Bdh2*-knockout ESCs, ESCs were used as control. Data were obtained in triplicate and presented as mean ± SD. *p*-values were calculated by two-way ANOVA, **p* < 0.05, ***p* < 0.001, ****p* < 0.0001. **(E)** Immunostaining of SOX2 and NANOG in *Bdh2*-knockout ESCs and ESCs. Scale bars, 50 μm. **(F)** Cell proliferation curves in *Bdh2*-knockout ESCs and ESCs. Data were obtained in triplicate and presented as mean ± SD. *p*-values were calculated by two-way ANOVA, **p* < 0.05, ***p* < 0.001, ****p* < 0.0001. **(G)** Cell cycle analysis of Bdh2-knockout ESCs and ESCs. The percentage of cells in G1, S, and G2 phase were determined by flow cytometry. Values are means of three independent experiments. Error bars indicate mean ± SD. **(H)** Real-time PCR analysis of endoderm, mesoderm and ectoderm-associated genes expression in the *Bdh2*-knockout ESCs, ESCs were used as control. Data were obtained in triplicate and presented as mean ± SD. *p*-values were calculated by two-way ANOVA, **p* < 0.05, ***p* < 0.001, ****p* < 0.0001. **(I)** Western blotting was used to detect the expression of BDH2, GATA4, and GATA6 in *Bdh2*-knockout ESCs and ESCs. **(J)** Immunostaining of GATA6 and SOX17 in *Bdh2*-knockout ASCs/ESCs and WT ASCs/ESCs. Scale bars, 50 μm.

Supplementary Figure 3 Molecular features of *Bdh2*-knockout ASCs. **(A)** Gene Ontology (GO) analysis of significantly enriched metabolism-related “biological processes” (BP) of the differentially expressed genes (DEGs) for Bdh2-knockout ASCs versus ASCs described in Figure 3A. **(B)** Heatmap showed up/down-regulated GO-BP terms and KEGG pathways, respectively. **(C)** Gene Set Enrichment Analysis (GSEA) analysis showing enriched GO-BP terms for up-regulated DEGs and down-regulated DEGs, respectively. **(D)** Heatmap listed a number of differentially expressed adhesion-related genes in Bdh2-knockout ASCs versus ASCs, enriched in three GO terms, “adhesion junction,” “focal adhesion,” and “gap junction.”

Supplementary Figure 4 *Bdh2* knockout regulates DNA methylation in ESCs. **(A)** Real-time PCR analysis of DNA methyltransferase genes expression (*Dnmt3a*, *Dnmt3b*, and *Dnmt3l*) in the *Bdh2*-knockout ESCs, ESCs were used as control. Data were obtained in triplicate and presented as mean ± SD. *p*-values were calculated by two-way ANOVA, **p* < 0.05, ***p* < 0.01, ****p* < 0.001. **(B)** Western blotting analysis for BDH2, DNMT3A, DNMT3B, DNMT3L, and DNMT1 in two *Bdh2*-knockout ESCs. **(C)** Immunostaining of DNMT3A in *Bdh2*-knockout ESCs and ESCs. Scale bars, 50 μm.

Supplementary Figure 5 Differentiated potency of *Bdh2*-knockout ESCs. **(A)** Real-time PCR analysis of endoderm, mesoderm and ectoderm-associated genes expression in the embryoid bodies of *Bdh2*-knockout ESCs, embryoid bodies of ESCs were used as control. Data were obtained in triplicate and presented as mean ± SD. *p* values were calculated by two-way ANOVA, **p* < 0.05, ***p* < 0.001, ****p* < 0.0001. **(B)** Morphology of embryoid bodies induction by *Bdh2*-knockout ASCs and ASCs in Day2, Day3, Day5, and Day7. Scale bars, 100 μm. **(C)** Morphology of embryoid bodies induction by *Bdh2*-knockout ESCs and ESCs in Day1∼Day7. Scale bars, 100 μm. **(D)** Statistics of the number and diameter of embryoid bodies formed by *Bdh2*-knockout ESCs and ESCs, respectively. N, represents the number of EB spheres. **(E)** Real-time PCR analysis of apoptotic and autophagy-associated genes expression in the embryoid bodies of *Bdh2*-knockout ESCs, embryoid bodies of ESCs were used as control. Data were obtained in triplicate and presented as mean ± SD. *p*-values were calculated by two-way ANOVA, **p* < 0.05, ***p* < 0.001, ****p* < 0.0001. **(F)** Representative bright-field images from *Bdh2*-knockout ESCs and ESCs differentiated for 5 days toward the primitive endoderm lineage. Scale bars, 50 μm. **(G)** Real-time PCR analysis of key markers after 4/5/6 days of directed differentiation toward primitive endoderm. Data were obtained in triplicate and presented as mean ± SD. *p*-values were calculated by two-way ANOVA, **p* < 0.05, ***p* < 0.001, ****p* < 0.0001.
