# Supplementary figures and images for: Bdh2 Deficiency Promotes Endoderm-Biased Early Differentiation of Mouse Embryonic Stem Cells
Source: Front Cell Dev Biol. 2021 Apr 8;9:655145. doi: 10.3389/fcell.2021.655145 (PMC8060705; doi:10.3389/fcell.2021.655145)

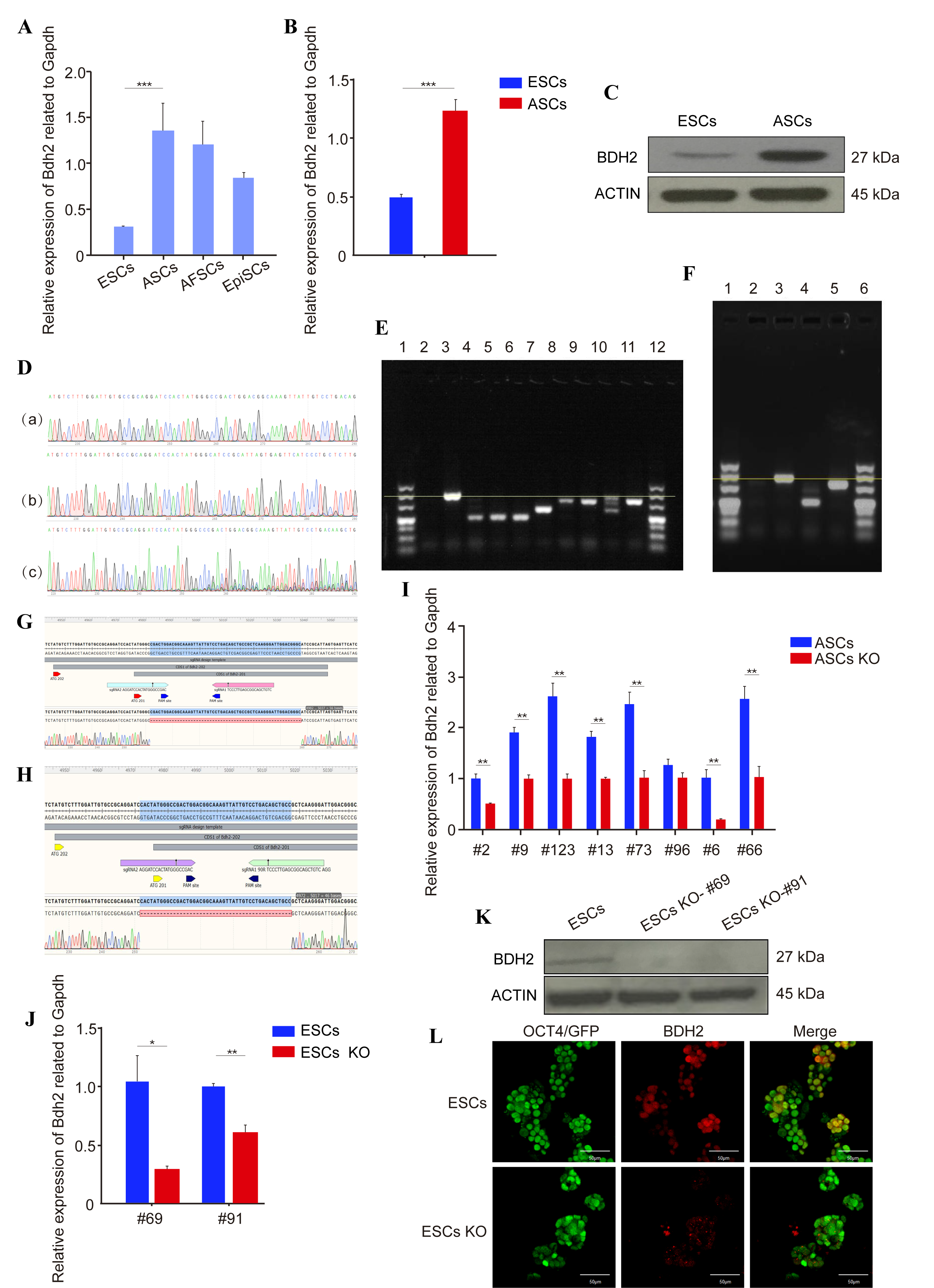

Supplement: Supplementary Figure 1 — Identification of bi-allelic Bdh2-knockout mouse embryonic stem cells (ESCs). (A) Expression of Bdh2 in different cell lines by RNA-Seq analysis. Data were obtained in triplicate and presented as mean ± SD. p-values were calculated by two-way ANOVA, *p < 0.05, **p < 0.001, ***p < 0.0001. (B) Real-time PCR analysis of Bdh2 expression in ESCs and ASCs. Data were obtained in triplicate and presented as mean ± SD. p-values were calculated by two-way ANOVA, *p < 0.05, **p < 0.001, ***p < 0.0001. (C) Western blotting was used to detect the expression of BDH2 in ESCs and ASCs. (D) The target region was sequenced to verify deletion in the desired region (a: wild type control; b: the sequence of bi-allelic knockout; c: the sequence of target region before single cell colony formation that showed overlapped pick in the gRNA site). (E) Genomic DNA PCR flanking the gRNAs sites from single cell colonies (1 and 12:DNA ladder; 2: Negative control; 3: Deletion not occurred; 4–9 and 11: Bi-allelic knockout; 10: Deletion occurred in one allele). (F) Genomic DNA PCR flanking the gRNAs sites from single cell colonies (1 and 6: DNA ladder; 2: Negative control; 3: Deletion not occurred; 4 and 5: Bi-allelic knockout). (G) Alignment between ASCs wild type sequence and bi-allelic knockout sequences that showed 56-nt deleted region. (H) Alignment between ESCs wild type sequence and bi-allelic knockout sequences that showed 46-nt deleted region. (I) Real-time PCR analysis of Bdh2 expression in the 7 bi-allelic knockout clones and a one-allelic knockout clone. Data were obtained in triplicate and presented as mean ± SD. p-values were calculated by two-way ANOVA, *p < 0.05, **p < 0.001, ***p < 0.0001. (J) Real-time PCR analysis of Bdh2 expression in the 2 bi-allelic knockout clones. Data were obtained in triplicate and presented as mean ± SD. p-values were calculated by two-way ANOVA, *p < 0.05, **p < 0.001, ***p < 0.0001. (K) Western blotting was used to detect the expression of BDH2 in the 2 [file Image_1.TIF]

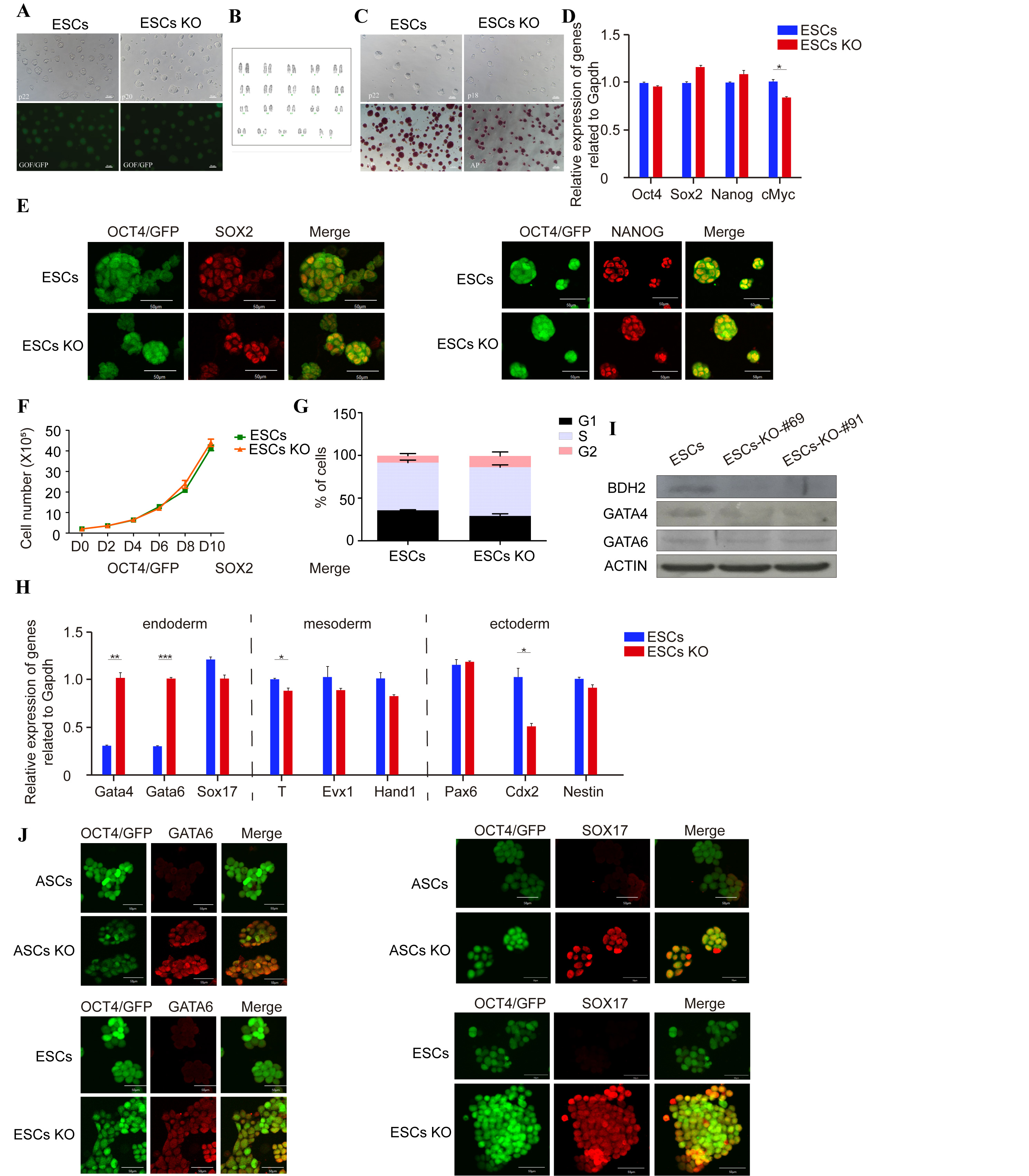

Supplement: Supplementary Figure 2 — Characteristics of Bdh2-knockout ESCs. (A) Morphology of Bdh2-knockout ESCs. Here, we use ESCs with GOF/GFP reporter. Scale bars, 50 μm. (B) Karyotyping of Bdh2-knockout ESCs (P25). (C) Alkaline phosphatase (AP) staining on Bdh2-knockout ESCs (P18) and ESCs (P22). Scale bars, 50 μm. (D) Real-time PCR analysis of pluripotency-associated genes expression in the Bdh2-knockout ESCs, ESCs were used as control. Data were obtained in triplicate and presented as mean ± SD. p-values were calculated by two-way ANOVA, *p < 0.05, **p < 0.001, ***p < 0.0001. (E) Immunostaining of SOX2 and NANOG in Bdh2-knockout ESCs and ESCs. Scale bars, 50 μm. (F) Cell proliferation curves in Bdh2-knockout ESCs and ESCs. Data were obtained in triplicate and presented as mean ± SD. p-values were calculated by two-way ANOVA, *p < 0.05, **p < 0.001, ***p < 0.0001. (G) Cell cycle analysis of Bdh2-knockout ESCs and ESCs. The percentage of cells in G1, S, and G2 phase were determined by flow cytometry. Values are means of three independent experiments. Error bars indicate mean ± SD. (H) Real-time PCR analysis of endoderm, mesoderm and ectoderm-associated genes expression in the Bdh2-knockout ESCs, ESCs were used as control. Data were obtained in triplicate and presented as mean ± SD. p-values were calculated by two-way ANOVA, *p < 0.05, **p < 0.001, ***p < 0.0001. (I) Western blotting was used to detect the expression of BDH2, GATA4, and GATA6 in Bdh2-knockout ESCs and ESCs. (J) Immunostaining of GATA6 and SOX17 in Bdh2-knockout ASCs/ESCs and WT ASCs/ESCs. Scale bars, 50 μm. [file Image_2.jpg]

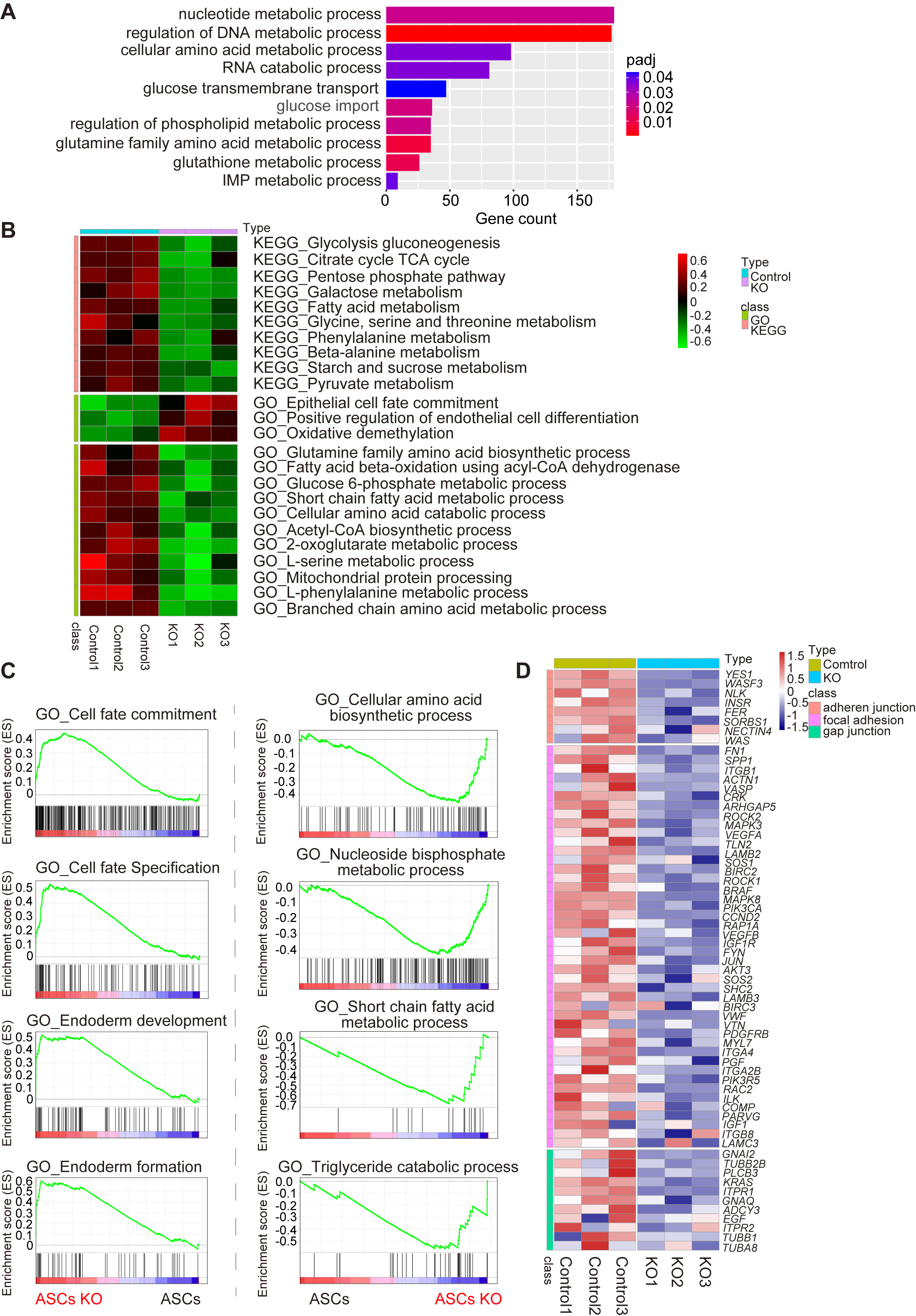

Supplement: Supplementary Figure 3 — Molecular features of Bdh2-knockout ASCs. (A) Gene Ontology (GO) analysis of significantly enriched metabolism-related “biological processes” (BP) of the differentially expressed genes (DEGs) for Bdh2-knockout ASCs versus ASCs described in Figure 3A. (B) Heatmap showed up/down-regulated GO-BP terms and KEGG pathways, respectively. (C) Gene Set Enrichment Analysis (GSEA) analysis showing enriched GO-BP terms for up-regulated DEGs and down-regulated DEGs, respectively. (D) Heatmap listed a number of differentially expressed adhesion-related genes in Bdh2-knockout ASCs versus ASCs, enriched in three GO terms, “adhesion junction,” “focal adhesion,” and “gap junction.” [file Image_3.JPEG]

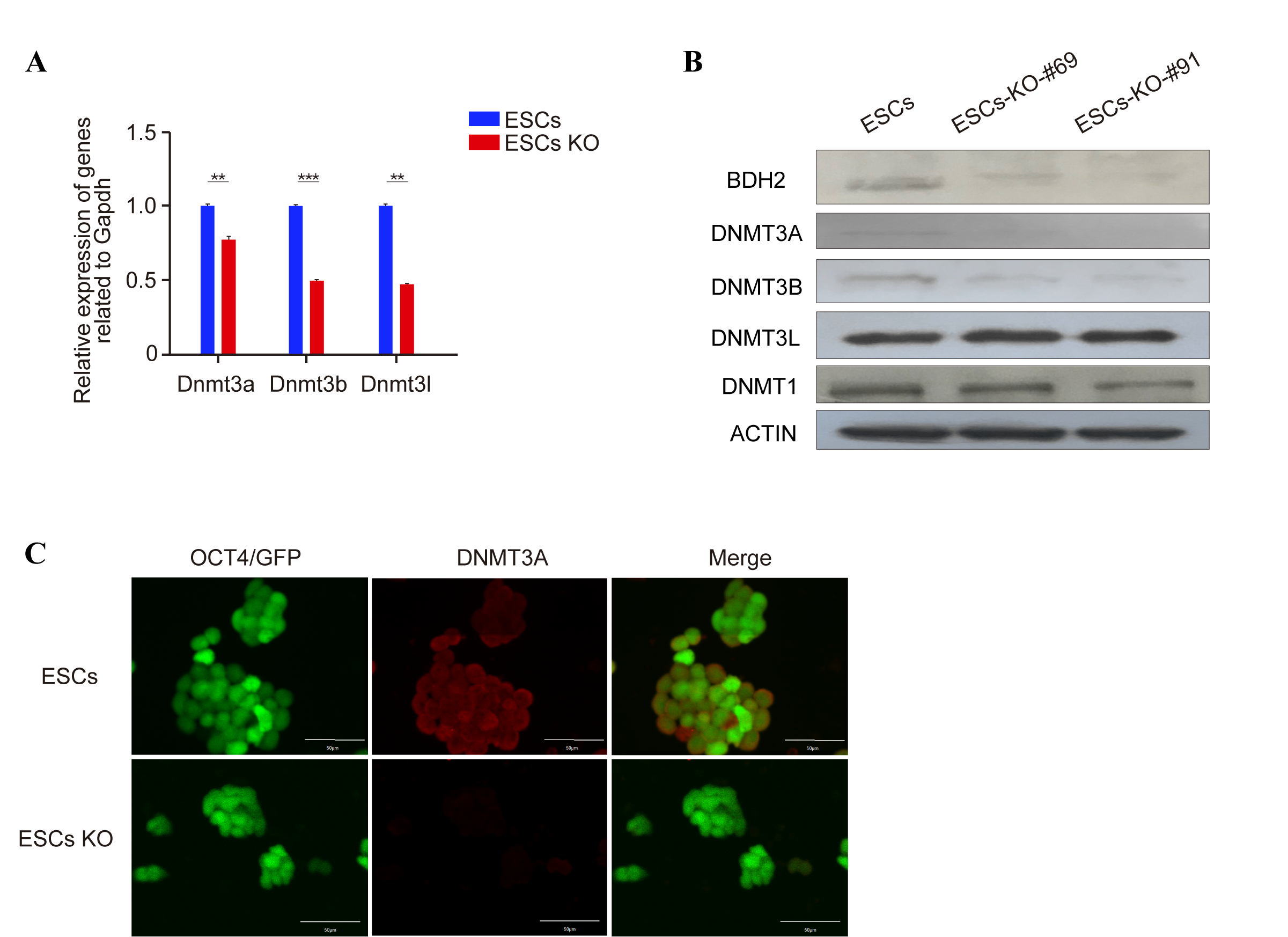

Supplement: Supplementary Figure 4 — Bdh2 knockout regulates DNA methylation in ESCs. (A) Real-time PCR analysis of DNA methyltransferase genes expression (Dnmt3a, Dnmt3b, and Dnmt3l) in the Bdh2-knockout ESCs, ESCs were used as control. Data were obtained in triplicate and presented as mean ± SD. p-values were calculated by two-way ANOVA, *p < 0.05, **p < 0.01, ***p < 0.001. (B) Western blotting analysis for BDH2, DNMT3A, DNMT3B, DNMT3L, and DNMT1 in two Bdh2-knockout ESCs. (C) Immunostaining of DNMT3A in Bdh2-knockout ESCs and ESCs. Scale bars, 50 μm. [file Image_4.TIF]
