# Supplementary material for: Bdh2 Deficiency Promotes Endoderm-Biased Early Differentiation of Mouse Embryonic Stem Cells
Source: Front Cell Dev Biol. 2021 Apr 8;9:655145. doi: 10.3389/fcell.2021.655145 (PMC8060705; doi:10.3389/fcell.2021.655145)
Supplement: Supplementary file 6 [file Data_Sheet_1.docx]

**Table S1 Guide RNA sequences**

| Gene name | sgRNA number | Sequence (5’-3’) | Base pair number (bp) |
| --- | --- | --- | --- |
| *Bdh2* | sgRNA1-Forward | CACCGTCCCTTGAGCGGCAGCTGTC | 25 |
|  | sgRNA1-Reverse | AAACGACAGCTGCCGCTCAAGGGAC | 25 |
|  | sgRNA2-Forward | CACCGAGGATCCACTATGGGCCGAC | 25 |
|  | sgRNA2-Reverse | AAACGTCGGCCCATAGTGGATCCTC | 25 |

**Table S2 Genotyping primers**

| Gene name | Sequence (5’-3’) | Base pair number (bp) |
| --- | --- | --- |
| *Bdh2*_4685F | AGCATTAGGATGCGACCGAG | 20 |
| *Bdh2*_5393R | TGGCAGGAGTGTGTGCTTAG | 20 |

**Table S3 The establishment rate of ASCs and ESCs**

| Cell line | Picked clone | Nucleotide deletion | Establishment rate |
| --- | --- | --- | --- |
| ASCs | 98 | 8 | 8.2 % |
| ESCs | 101 | 2 | 2 % |

**Table S4 RT-qPCR primers**

| Gene name | Forward primer (5’-3’) | Reverse primer (5’-3’) |
| --- | --- | --- |
| *Bdh2* | GAAAGAATACAAGCCAGAG | GACAGGGTTGCCAGTTA |
| *Oct4* | GCTTGGGCTAGAGAAGGATGTG | TGGCGCCGGTTACAGAAC |
| *Sox2* | CATGAGAGCAAGTACTGGCAAG | CCAACGATATCAACCTGCATGG |
| *Nanog* | AAACCAGTGGTTGAAGACTAGCAA | GGTGCTGAGCCCTTCTGAATC |
| *Gata4* | TTCCTCTCCCAGGAACATCAAA | GCTGCACAACTGGGCTCTACTT |
| *Gata6* | TGCTGGAAATTGCAACAAACC | GTCACGTGGTACAGGCGTCA |
| *Sox17* | GTCAACGCCTTCCAAGACTTG | GTAAAGGTGAAAGGCGAGGTG |
| *T* | GGACCTCGGATTCACATCGT | TTCTTTGGCATCAAGGAAGG |
| *Evx1* | CCAGTGACCAGATGCGCCGATAC | TCCTTCATGCGCCGGTTCT |
| *Hand1* | TCAAAAAGACGGATGGTGGT | GCGCCCTTTAATCCTCTTCT |
| *Cdx2* | CCTGCGACAAGGGCTTGTTTAG | TCCCGACTTCCCTTCACCATAC |
| *Pax6* | GCAGATGCAAAAGTCCAGGTG | CAGGTTGCGAAGAACTCTGTTT |
| *Nestin* | CTCGAGCAGGAAGTGGTAGG | TTGGGACCAGGGACTGTTAG |
| *Dnmt3a* | GACTCGCGTGCAATAACCTTAG | GGTCACTTTCCCTCACTCTGG |
| *Dnmt3b* | CTCGCAAGGTGTGGGCTTTTGTAAC | CTGGGCATCTGTCATCTTTGCACC |
| *Dnmt3l* | CGGAGCATTGAAGACATC | CATCATCATACAGGAAGAGG |
| *cMyc* | GACTCTGAAGAAGAGCAAGAAGATGA | TCCACAGACACCACATCAATTTC |
| *Gapdh* | ACCACAGTCCATGCCATCAC | TCCACCACCCTGTTGCTGTA |

**Table S5 Antibody information**

| **Reagent** | **Source** | **Identifier** | **Dilution Ratio** |
| --- | --- | --- | --- |
| **Antibodies for IF** | | |  |
| BDH2 | Proteintech | 27207-1-AP | 1:200 |
| SOX2 | Santa cruz | Sc-17320 | 1:200 |
| NANOG | eBioscience | 14-5761 | 1:500 |
| GATA4 | Millipore | 07-449 | 1:500 |
| GATA6 | R＆D systems | MAB1700 | 1:100 |
| T | R＆D systems | MAB20851 | 1:100 |
| NESTIN | R＆D systems | MAB2736 | 1:200 |
| Alexa Fluor 405 donkey anti-mouse lgG | invitrogen | A21202 | 1:500 |
| Alexa Fluor 405 donkey anti-goat lgG | invitrogen | A11055 | 1:500 |
| Alexa Fluor 405 donkey anti-rat lgG | invitrogen | A21208 | 1:500 |
| **Antibodies for WB** |  |  |  |
| Rabbit anti-β-ACTIN | Cell signaling | 5125 | 1:1000 |
| BDH2 | Cell signaling | D3A7 | 1:1000 |
| GATA4 | Millipore | 07-449 | 1:1000 |
| GATA6 | R＆D systems | MAB1700 |  |
| DNMT3A | Abcam | ab188470 | 1:2000 |
| DNMT3B | Abcam | ab119282 | 1:500 |
| DNMT3L | Abcam | ab194094 | 1:1000 |
| DNMT31 | Abcam | ab188453 | 1:1000 |
| p-mTOR | Cell signaling | #5536s | 1:1000 |
| mTOR | Cell signaling | #2983 | 1:1000 |
| p-S6 | Cell signaling | #4858 | 1:2000 |
| S6 | Cell signaling | #2317s | 1:1000 |
| p-4EBP1 | Cell signaling | #2855 | 1:1000 |
| 4EBP1 | Cell signaling | #9644 | 1:1000 |
| Rabbit anti-Goat IgG-HRP | Absin | Abs20005 | 1:5000 |
| Goat anti-rabbit IgG-HRP | Absin | Abs20002A | 1:5000 |
| Goat anti-mouse IgG-HRP | Absin | abs20001A | 1:5000 |
